# Revisiting Late‐Onset Bipolar Disorder Associated With Pituitary Adenoma: A Cross‐Sectional, Multidisciplinary, Case‐Based Analysis

**DOI:** 10.1002/brb3.70611

**Published:** 2025-06-10

**Authors:** Yagmur Sever Fidan, Ikbal Humay Arman, Sumeyye Yasemin Calli, Fusun Mayda Domac, Emine Nese Yeniceri, Bahar Tasdelen, Aynur Ozge

**Affiliations:** ^1^ Erenkoy Mental and Nervous Diseases Training and Research Hospital Istanbul Turkiye; ^2^ Faculty of Medicine, Department of Family Medicine Istanbul Medipol University Istanbul Turkiye; ^3^ Faculty of Medicine, Department of Family Medicine Mugla Sitki Kocman University Mugla Turkiye; ^4^ Faculty of Medicine, Department of Biostatistics and Medical Informatics Mersin University Mersin Turkiye

**Keywords:** diagnostic challenges, late‐onset bipolar disorder, neuroendocrine disorders, pituitary adenoma

## Abstract

**Objective:**

This study explores the diagnostic, treatment, and follow‐up approaches of neurologists, psychiatrists, and family physicians in managing late‐onset bipolar disorder (BD) in elderly patients, emphasizing the role of interdisciplinary collaboration.

**Methods:**

A cross‐sectional survey involved 300 specialists (100 from each discipline). The survey assessed diagnostic accuracy, treatment preferences, follow‐up adherence, and barriers to interdisciplinary collaboration. A pairwise *z*‐test with Bonferroni correction was applied for comparative analysis across specialties to evaluate differences in response proportions.

**Results:**

Neurologists demonstrated high proficiency in imaging and treatment initiation (91% correct imaging responses) but faced challenges in holistic management and follow‐up adherence (2%). Psychiatrists excelled in diagnosing organic causes of mood disorders (92%) and therapeutic decisions but struggled with non‐priority diagnostic tests (44%) and long‐term follow‐up strategies (14%). Family physicians showed strong skills in pharmacological management (96%) and follow‐up adherence (89%) but encountered barriers like stigma and referral timing for high‐risk cases. Common interdisciplinary challenges included diagnostic communication, treatment coordination, and follow‐up collaboration.

**Conclusion:**

Late‐onset BD management requires a multidisciplinary approach to address specialty‐specific gaps and foster effective interdisciplinary collaboration. Enhanced training, integrated care models, and shared guidelines are recommended to optimize outcomes for elderly patients with mood disorders. Future research should focus on developing standardized protocols and evaluating the long‐term impact of interdisciplinary interventions.

## Graphical abstract

Surveying 300 neurologists, psychiatrists, and family physicians, this study demonstrates discipline‐specific strengths‐imaging (neurology), diagnosis (psychiatry), and pharmacological follow‐up (family medicine)‐yet persistent gaps in holistic care of late‐onset bipolar disorder in elders. Shared guidelines and integrated, cross‐disciplinary training are essential to bridge gaps and improve outcomes for elderly patients.

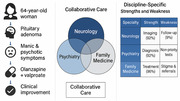

## 1 Introduction

Bipolar disorder (BD) is a chronic mental health condition characterized by alternating episodes of mania and depression, leading to significant morbidity, mortality, and a profound impact on quality of life. Globally, BD affects approximately 1%–4% of the population, with varying prevalence based on demographic and diagnostic criteria. (Charney et al. 2020). While BD is commonly associated with late adolescence or early adulthood, a substantial proportion of patients experience the onset later in life, a phenomenon often overlooked in clinical practice. Studies suggest that the prevalence of BD in the geriatric population may be underestimated due to diagnostic challenges and overlapping presentations with other age‐related conditions (Chancel et al. 2024).

The age of onset in BD is a critical factor that influences clinical presentation and treatment strategies. Late‐onset BD is frequently associated with organic causes, such as neuroendocrine dysfunctions or structural brain abnormalities, including pituitary adenomas. These findings highlight the importance of distinguishing between early‐ and late‐onset BD to tailor interventions effectively (Almeida et al. 2018).

The management of elder‐onset BD presents unique challenges that require a multidisciplinary approach. Family physicians often serve as the initial point of contact, providing continuity of care and identifying early symptoms. Neurology clinics frequently address the neurobiological and structural contributors to late‐onset mood disorders, while psychiatric consultations, although critical, often occur at advanced stages of the condition. This fragmentation underscores the need for integrated care models to optimize outcomes for elderly patients (Özge et al. 2023).

Informed by the case vignette described in this study, the questionnaire items were designed to reflect key diagnostic, therapeutic, and follow‐up challenges that specialists may encounter in clinical settings. For example, the patient's prolonged refusal of treatment, absence of psychiatric follow‐up, and eventual diagnosis of a pituitary macroadenoma served as guiding points to explore how different specialties approach late‐onset bipolar symptoms, particularly when secondary to organic causes. This alignment allowed for the capture of real‐world attitudes and practices, thereby enhancing the survey's clinical relevance and ecological validity.

This questionnaire‐based study investigates the diagnostic approaches, management strategies, and coping mechanisms employed by neurology, psychiatry, and family medicine specialists for elderly‐onset BD. By surveying 100 specialists from each discipline, this research seeks to identify prominent practices and gaps within each specialty and explore potential interdisciplinary differences. Furthermore, the study highlights the importance of multidisciplinary collaboration in addressing the complex needs of elderly patients with BD, with the ultimate goal of enhancing patient care and improving clinical outcomes.

### 1.1 Case

This case elaborates on the management of a 64‐year‐old female patient brought to the hospital by her relatives to evaluate the necessity of guardianship due to her long‐standing refusal of treatment despite episodic complaints over approximately 10 years. For the past two months, her symptoms had significantly worsened, prompting her relatives to seek medical evaluation. The patient had not previously sought psychiatric consultation and presented with irritability, racing thoughts, excessive talking, insomnia, increased goal‐directed activity, disheveled appearance, heightened energy, and accompanying paranoid psychotic symptoms.

Upon admission to the hospital's medical board department, the patient underwent a comprehensive evaluation, including laboratory tests, cranial imaging, and EEG, to investigate the etiology of her mood disorder symptoms. Additional assessments included a neuropsychiatric test battery (NPT), a Mini‐Mental State Examination (MMSE), and a Rorschach test. The Young Mania Rating Scale (YMRS) score at admission was 37, indicating severe manic symptoms. Laboratory findings revealed no abnormalities. NPT results indicated “mild impairment related to the frontal lobe and memory impairment.” The MMSE yielded a score of 26 out of 30, suggesting mild cognitive deficits. The EEG findings were within normal limits.

Cranial MRI identified a lesion in the pituitary gland measuring 20 × 16 mm, characterized by heterogeneous contrast enhancement in contrast‐enhanced sections with mild hypointensity on T1 and T2 weighted sequences, indicative of a macroadenoma. Subsequent consultations with endocrinology and ophthalmology were arranged for further evaluation. Hormonal assays were conducted, confirming the diagnosis of a non‐functioning pituitary macroadenoma. A visual field examination was attempted but could not be completed due to the patient's inability to cooperate. A consultation with the neurosurgery department ruled out the need for urgent surgical intervention.

During admission to the psychiatric service, pharmacological treatment was initiated and titrated to address the patient's psychiatric symptoms. Valproic acid was prescribed at a dosage of 750 mg/day, and olanzapine was initiated at 7.5 mg/day. By the third week of admission, the patient's YMRS score had decreased to 10, reflecting significant symptom improvement.

This case highlights the chronic nature of the disorder and the patient's longstanding treatment refusal, which culminated in her seeking medical attention only after severe psychiatric symptoms emerged due to the pituitary adenoma. The necessity of a multidisciplinary approach is evident, with collaboration across specialties—including psychiatry, endocrinology, neurology, and neurosurgery—to ensure timely diagnosis and intervention. This case underscores the importance of proactive, integrated care to address psychiatric, neuroendocrine, and functional impairments before they lead to substantial social and functional deterioration.

## 2 Method

Study Design: This study adopts a cross‐sectional, questionnaire‐based design to assess the perspectives and practices of neurology, psychiatry, and family medicine specialists in diagnosing, managing, and coping with strategies for late‐onset mood disorders.

Participants: This study recruited 300 specialists, including 100 neurologists, 100 psychiatrists, and 100 family physicians. Participants were selected based on their active clinical practice and expertise in managing late‐onset mood disorder patients.

Questionnaire Development: Based on the case study provided and a comprehensive review of relevant literature, three structured questionnaires were developed, each tailored to the specific expertise and perspectives of neurologists, psychiatrists, and family physicians.

The questionnaires included items addressing:

· Diagnostic approaches, including imaging, laboratory tests, and symptom recognition specific to late‐onset mood disorders and associated conditions such as pituitary adenomas.
· Treatment modalities focus on pharmacological preferences, management of comorbidities, and understanding complications of specific interventions.
· Follow‐up practices, including strategies for adherence monitoring and addressing barriers to long‐term care.
· Interdisciplinary collaboration to assess specialists’ attitudes toward team‐based approaches and perceived barriers to optimal patient care.

Each questionnaire was designed to capture discipline‐specific challenges and strengths while facilitating comparative analysis across specialties. Key themes included mental health stigma, neuroendocrine‐psychiatric integration, and models of collaborative care. The development process also included pilot testing with a small group of specialists to refine questions for clarity and relevance.

Questionnaire Validation: In order to guarantee content validity, the items of the questionnaire were established in accordance with evidence‐based clinical guidelines and up‐to‐date literature (Yatham et al. 2018; Arnold et al. 2021; Lane and Smith 2023). A subsequent review of the items was carried out by an expert panel composed of two psychiatrists, two neurologists, and two family physicians. The members of the panel had clinical and academic experience in managing late‐onset mood disorders. Each expert independently evaluated the items' relevance, clarity, and representativeness. Based on their feedback, minor revisions were made to enhance item precision and reduce ambiguity. Although formal psychometric testing was beyond the scope of this study, the questionnaire's construct validity was preliminarily addressed through pilot testing and expert review. Feedback obtained during the pilot phase led to revisions that improved clarity and construct alignment.

Data Collection: The questionnaire was administered electronically via a secure online platform (e.g., Google Forms). Specialists were invited via email to participate in the study and complete the questionnaire anonymously. Participation was voluntary, and informed consent was obtained from all participants before data collection.

### 2.1 Ethical Considerations

This study adhered to the ethical guidelines outlined by relevant institutional review boards. Participants' anonymity and confidentiality were ensured throughout the study process. Informed consent was obtained from all participants, and they had the right to withdraw from the study at any time without penalty.

### 2.2 Risk of Bias Assessment

To mitigate potential sources of bias in this study, several strategies were implemented:

1. Sampling Bias: Efforts were made to recruit a diverse and representative sample of specialists from each discipline. Participants were selected from various geographical locations and practice settings to enhance the generalizability of the findings.
2. Non‐Response Bias: To minimize non‐response bias, reminders were sent to invited specialists who had not yet completed the questionnaire. In addition, the anonymity of responses was emphasized to encourage candid feedback.
3. Measurement Bias: The questionnaire items were carefully constructed to ensure clarity and relevance to the study objectives. Pilot testing was conducted with a small group of specialists to assess the questionnaire's comprehensibility and appropriateness.
4. Recall Bias: Participants were asked to provide information based on their clinical experiences and practices. To minimize recall bias, questions were framed in a way that elicited recent and relevant experiences rather than relying on distant memories.
5. Reporting Bias: All collected data were analyzed and reported transparently, including significant findings and null results. The study report acknowledged and discussed any discrepancies or inconsistencies in responses.
6. Conflict of Interest: Potential conflicts of interest among participants, such as financial relationships with pharmaceutical companies, were disclosed. Participants were asked to refrain from providing biased responses influenced by personal or professional interests.
7. Interdisciplinary Bias: Given the interdisciplinary nature of the study, efforts were made to maintain neutrality and impartiality in interpreting findings across different medical specialties. Any observed differences or conflicts between specialties were analyzed objectively and discussed in the context of interdisciplinary collaboration.

By addressing these potential sources of bias, this study aimed to enhance the validity and reliability of its findings, ultimately contributing to a more robust understanding of late‐onset mood disorder management across neurology, psychiatry, and family medicine specialties.

### 2.3 Data Analysis

Quantitative data collected from the questionnaire responses were analyzed using descriptive statistics, including frequencies and percentages. For comparative analysis across specialties, pairwise *z*‐tests were applied to evaluate differences in response proportions. Bonferroni correction was applied for the number of tests within each row (for k columns, *p*_bonf_ = p.k(k‐1)/2). Statistical significance was set at *p* < 0.05.

The response ratios for correct answers were calculated and compared between specialties to examine specific patterns in diagnostic accuracy, treatment strategies, and follow‐up adherence. Qualitative responses from open‐ended questions were subjected to thematic analysis. Key themes and patterns were identified through iterative coding, focusing on barriers to interdisciplinary collaboration, variations in clinical judgment, and shared challenges in managing mood disorders in elderly patients.

## 3 Results

**FIGURE 1:**
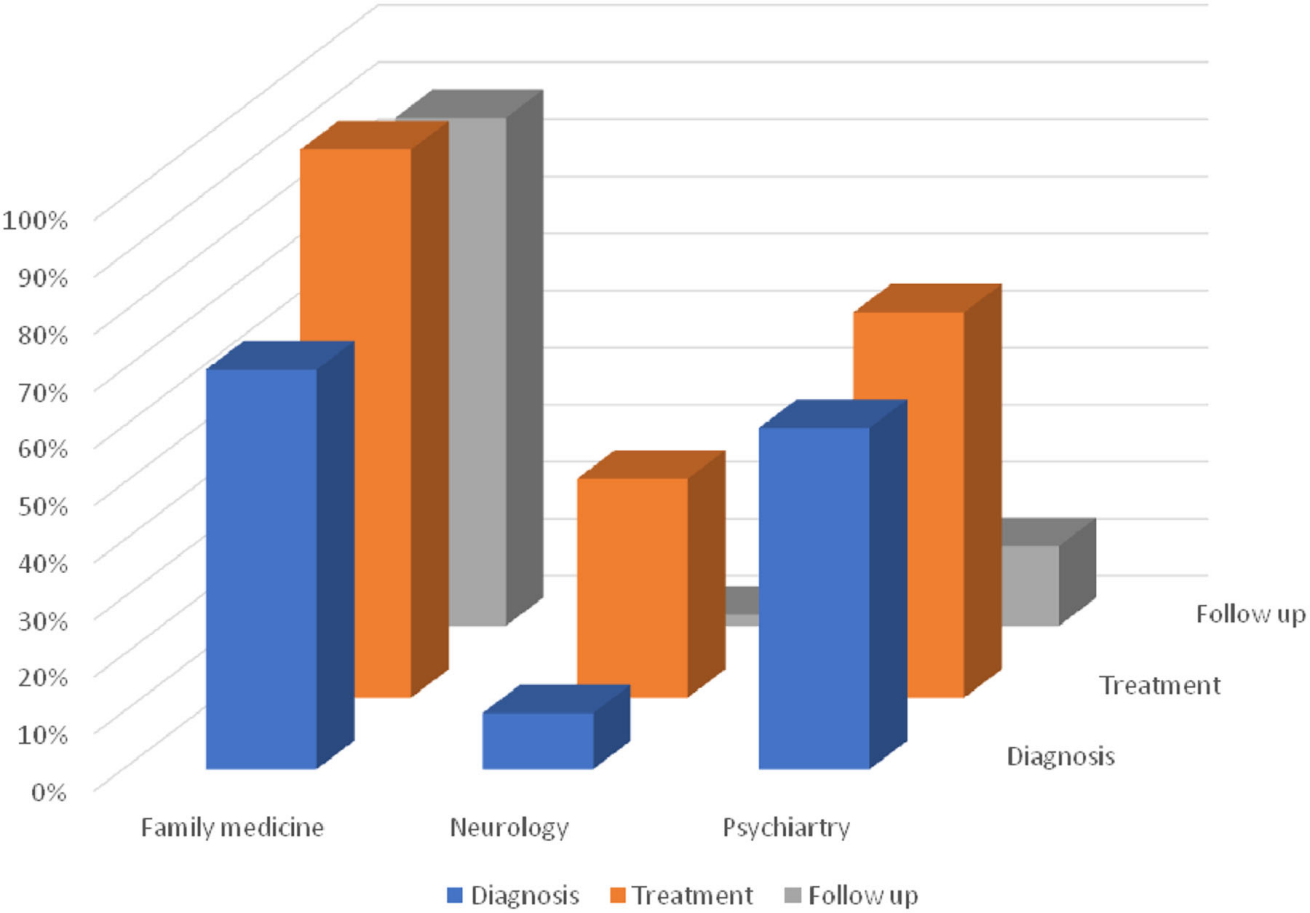
The distribution of correct responses across specialties in diagnosis, treatment, and follow‐up.

This study systematically evaluates the diagnostic accuracy, treatment strategies, and follow‐up practices employed by neurologists, psychiatrists, and family physicians in managing late‐onset mood disorders, specifically focusing on interdisciplinary collaboration. Around 300 specialists participated, providing responses highlighting notable variations in practice patterns, strengths, and challenges across disciplines. While neurologists excelled in imaging and initial treatment protocols, their adherence to comprehensive follow‐up plans was markedly low. Psychiatrists demonstrated high proficiency in diagnosing mood disorders with organic causes and selecting appropriate therapeutic interventions, yet struggled with non‐priority diagnostic tests and sustained follow‐up practices. Family physicians, often the first point of contact for elderly patients, showed strong capabilities in pharmacological management and treatment adherence but faced systemic barriers, including stigma and limited resources for interdisciplinary coordination. These findings underscore the multifaceted challenges and the critical need for integrated care models in managing mood disorders among elderly populations (see Table 1 and Figure 1).

**TABLE 1:** The response proportions of correct answers for diagnosis, treatment, and follow‐up questions in each clinic.

The response ratios of accurate answers	Family medicine	Neurology	Psychiatry
**Diagnosis**	70%^a^	10%^b^	60%^a^
**Treatment**	96%^a^	38%^b^	68%^c^
**Follow up**	89%^a^	2%^b^	14%^c^

*Note*: The different subscript letters denote whose column proportions differ significantly in each row.

Table 1 illustrates the response ratios of correct answers across diagnostic, treatment, and follow‐up questions for each specialty. The data reveal significant variability in expertise areas: family physicians excelled in treatment (96%) and follow‐up (89%), highlighting their strength in patient management and continuity of care. Neurologists demonstrated notable challenges in follow‐up (2%) but showed relatively higher accuracy in treatment (38%), reflecting their role in acute management rather than longitudinal care. Psychiatrists exhibited balanced performance, focusing strongly on treatment (68%) and diagnosis (60%), indicating their comprehensive understanding of mood disorders. These differences underscore the importance of interdisciplinary collaboration to bridge gaps in care.

It is observed that there is no statistical difference between family medicines and psychiatrists for correct answers in diagnosis questionaries (60%–70%, *p*_bonf_ = 0.416). In contrast, neurologists are statistically significantly different from the others (^a‐b,b‐c^: *p*_bonf_ **< 0.001). All categories are different for correct answers in treatment questionaries (^a‐b,b‐c,a‐c^:*p*_bonf _< 0.001). Similarly, all categories differ for correct answers in follow‐up questionnaires (^a‐b,a‐c^:*p*_bonf _< 0.001, ^b‐c^: *p*_bonf_ = 0.005).

Figure 1 visualizes the distribution of correct responses, emphasizing the disparities among specialties. Neurologists' and psychiatrists' low follow‐up adherence contrasts sharply with family physicians' strong performance in this domain, suggesting that systematic frameworks for follow‐up could enhance care integration. The complementary strengths depicted in the figure further advocate for a multidisciplinary approach to optimizing patient outcomes in late‐onset mood disorders.

### 3.1 Neurologist Survey Results

The survey responses from neurologists highlight key trends and challenges in diagnosing, treating, and monitoring late‐onset mood disorders. The response ratios for correct answers across the diagnostic, treatment, and follow‐up domains varied. Detailed analysis of individual survey questions revealed:

1. Diagnostic Accuracy:
  ○ The highest accuracy was observed in questions related to imaging findings of pituitary adenomas (91%).
  ○ Notable gaps were identified in recognizing hormonal markers and differentiating pituitary apoplexy from other conditions, with correct response rates of 5% and 24%, respectively.
2. Treatment Strategies:
  ○ A significant proportion (77%) correctly identified the first‐line treatment for functional prolactinomas.
  ○ Challenges were noted in understanding psychiatric complications related to bromocriptine treatment, with a 38% correct response rate.
3. Follow‐Up Practices:
  ○ The most correctly identified follow‐up issue was the type of visual field defect commonly associated with pituitary adenomas (90%).
  ○ Adherence to comprehensive follow‐up protocols remained a significant area for improvement (2%).

The variability in correct responses underscores the need for targeted education and training in specific aspects of mood disorder management. A comparison with responses from psychiatry and family medicine revealed interdisciplinary gaps that may hinder collaborative care.

These findings suggest that while neurologists demonstrate proficiency in areas related to imaging and treatment initiation, challenges persist in providing holistic management and integrating interdisciplinary approaches. Future efforts should focus on addressing these gaps to improve outcomes for patients with late‐onset mood disorders.

These findings suggest that while neurologists demonstrate proficiency in imaging and treatment initiation areas, challenges persist in holistic management and interdisciplinary integration. Future efforts should focus on addressing these gaps to improve outcomes for patients with late‐onset mood disorders.

### 3.2 Psychiatrist Survey Results

Psychiatrist' responses offered valuable insights into the management of mood disorders with organic causes, highlighting their expertise in diagnostic and therapeutic decision‐making. Detailed analysis of individual survey questions revealed:

1. Diagnostic Insights:
  ○ High accuracy was observed in identifying the most common organic causes of mood disorders, with 92% correctly recognizing prolactinomas as the predominant type.
  ○ Challenges were noted in distinguishing non‐priority diagnostic tests, such as cranial tomography, with a correct response rate of 44%.
2. Treatment Knowledge:
  ○ Respondents displayed proficiency in selecting appropriate antipsychotics for prolactinoma cases, with aripiprazole being correctly identified by 78% of participants.
  ○ A notable gap was observed in recognizing the mood disorder symptoms associated with medications like bromocriptine (31%).
3. Follow‐Up Practices:
  ○ The least accuracy was observed in follow‐up strategies, with only 14% of psychiatrists identifying correct practices for managing patients with mood symptoms due to functional adenomas.

These findings indicate that psychiatrists excel in areas requiring an in‐depth understanding of mood disorders but face challenges in follow‐up protocols, particularly in interdisciplinary contexts. Addressing these gaps through enhanced training and collaboration may optimize patient outcomes.

### 3.3 Family Physician Survey Results

Family physicians demonstrated strong foundational skills in diagnosing and managing late‐onset mood disorders, with notable areas for improvement in interdisciplinary collaboration and addressing barriers in mental health care. Detailed analysis of individual survey questions revealed:

1. Diagnostic Accuracy:
  ○ Strong performance was observed in recognizing the typical age group for BD onset (74%) and distinguishing it from conditions like ADHD or eating disorders (79%).
  ○ Some challenges remained in addressing stigma and misconceptions about mental health in elderly populations.
2. Treatment Strategies:
  ○ Family physicians excelled in managing pharmacological treatments for elderly patients, with 96% accuracy in addressing drug‐drug interactions and adverse effects.
  ○ Referral practices showed room for improvement, particularly in identifying high‐risk cases warranting psychiatric consultation.
3. Follow‐Up Practices:
  ○ High adherence to follow‐up protocols was evident, with 89% accuracy in identifying strategies to monitor depression's impact on medical comorbidities and treatment adherence.
  ○ Gaps were noted in addressing barriers such as stigma and age‐related misconceptions.

These findings highlight the essential role of family physicians in managing mood disorders at the primary care level. Addressing gaps in stigma reduction, referral timing, and interdisciplinary collaboration will further enhance their ability to provide comprehensive care.

Table 2 summarizes the key challenges neurologists, psychiatrists, and family physicians face in diagnosis, treatment, and follow‐up.

**TABLE 2:** Interdisciplinary comparisons of the main key challenges.

Aspect	Neurologists	Psychiatrists	Family physicians
**Diagnosis**	‐ Low accuracy in identifying hormonal markers (5%).	‐ Difficulty in recognizing non‐priority diagnostic tests (44%).	‐ Challenges distinguishing bipolar symptoms from overlapping conditions like ADHD (79%).
	‐ Difficulty differentiating pituitary apoplexy from other conditions (24%).	‐ Gaps in identifying some organic causes of mood disorders.	‐ Barriers due to stigma and misconceptions about mental health in elderly populations.
**Treatment**	‐ Limited understanding of psychiatric complications from bromocriptine (38%).	‐ Gaps in recognizing mood disorder symptoms linked to medications like bromocriptine (31%).	‐ Challenges in timely referrals for high‐risk cases, such as suicidality.
	‐ Inadequate interdisciplinary collaboration for holistic treatment.	‐ Gaps in integrating endocrinological or neurological aspects into psychiatric care plans.	‐ Need for greater emphasis on lifestyle interventions alongside pharmacological treatments.
**Follow‐up**	‐ Minimal adherence to follow‐up protocols (2%).	‐ Limited recognition of effective follow‐up strategies for functional adenomas (14%).	‐ Gaps in addressing systemic barriers like stigma during follow‐up (89% adherence otherwise).
	‐ Lack of comprehensive strategies for long‐term monitoring of outcomes.	‐ Poor collaboration with other specialties during follow‐up phases.	‐ Need for stronger interdisciplinary frameworks for follow‐up and long‐term care coordination.

*Note*: Each row highlights specific gaps or limitations observed within each specialty, reflecting areas where improvements or interdisciplinary collaboration could enhance patient care outcomes. The percentages in parentheses indicate the correct response ratios from survey data, where applicable, illustrating the extent of the challenges.

**FIGURE 2:**
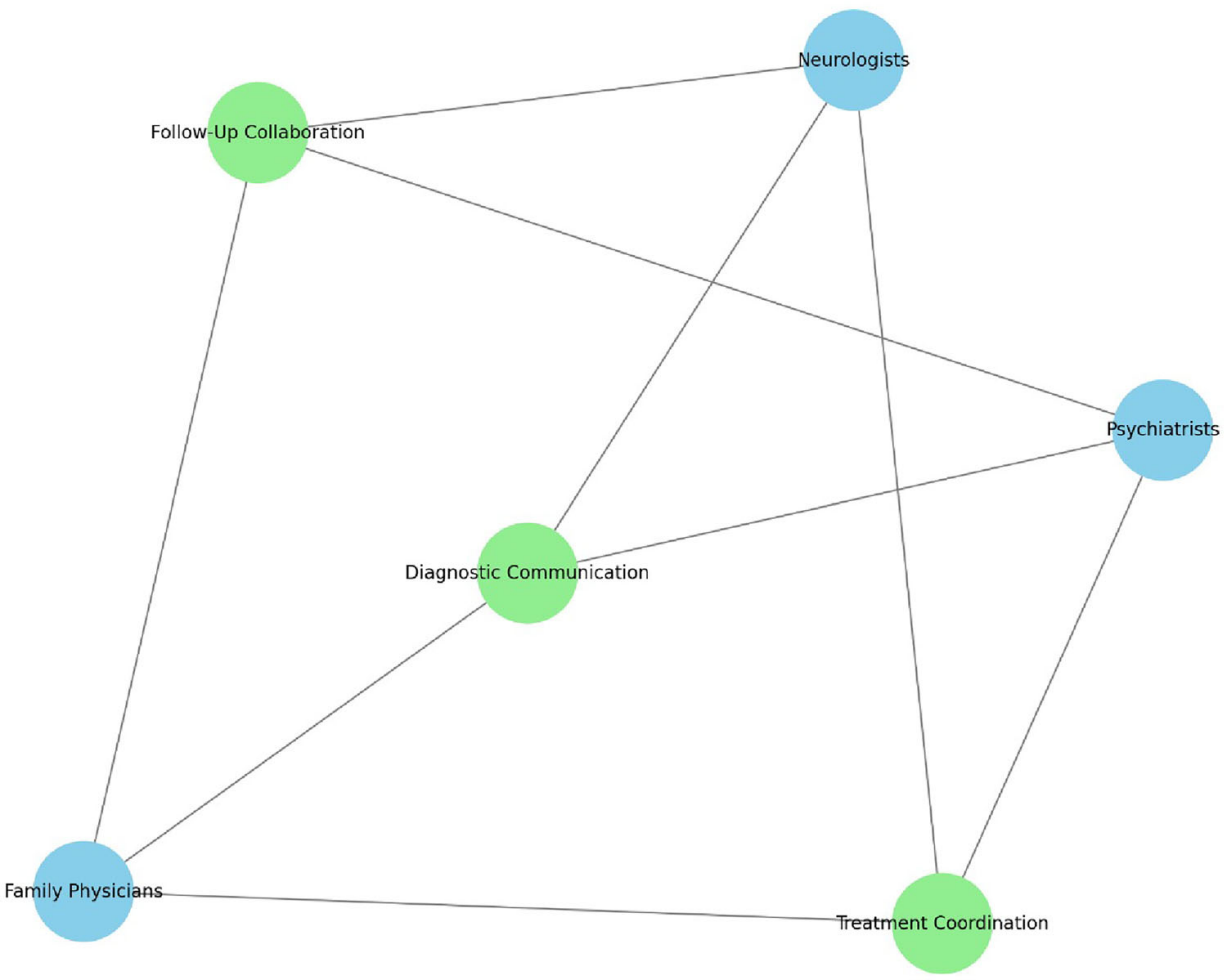
Network of perceived barriers to interdisciplinary collaboration among specialties.

In Figure 2, the network diagram represents the challenges of interdisciplinary collaboration. Nodes represent specialties (e.g., neurologists, psychiatrists, family physicians) and shared challenges (diagnostic communication, treatment coordination, and follow‐up collaboration). Edges highlight connections based on shared challenges.

## 4 Discussion

Managing late‐onset BD in geriatric patients requires a nuanced understanding of psychiatric and neurological factors. This discussion incorporates additional references to provide a comprehensive overview of this area's current knowledge and practice.

### 4.1 Understanding Late‐Onset BD

Late‐onset BD, typically defined as BD manifesting after the age of 60, raises unique diagnostic and therapeutic challenges. Research indicates that the prevalence of BD in older adults is often underestimated, with some studies suggesting that up to 5% of the elderly population may be affected by mood disorders, including BD (Lenzenweger et al. 2007).

The differentiation between early‐ and late‐onset BD is crucial, as late‐onset cases are frequently associated with identifiable organic causes, such as neuroendocrine disorders, including pituitary adenomas. Recent studies have highlighted the importance of recognizing the trimodal distribution of BD onset, which includes early, middle, and late phases. This distribution suggests that while early‐onset BD is more common, late‐onset cases may present differently and require distinct management strategies. Notably, a meta‐analysis has revealed that late‐onset BD is often associated with a higher incidence of comorbid medical conditions, underscoring the need for comprehensive evaluations (Lane and Smith 2023).

### 4.2 Interdisciplinary Collaboration

The complexity of managing late‐onset BD necessitates collaboration among neurologists, psychiatrists, and family physicians. Each specialty contributes unique insights into the diagnosis and treatment process. Neurologists are adept at identifying neuroanatomical abnormalities through imaging techniques, while psychiatrists focus on the psychological aspects and therapeutic interventions. Family physicians often serve as patients' initial point of contact, making their role critical in recognizing symptoms and facilitating timely referrals (Hawke et al. 2013). Studies highlight the importance of interdisciplinary approaches in managing complex cases where mood disorders intersect with neurological conditions. The authors advocate for integrated care models that promote communication among specialists to enhance patient outcomes. Furthermore, collaborative care has been shown to improve adherence to treatment protocols among elderly patients with mood disorders (Arnold et al. 2021)

In this context, digital health tools offer a promising means of enhancing interdisciplinary coordination and addressing follow‐up gaps. Shared electronic health records enable seamless information flow between neurologists, psychiatrists, and family physicians, ensuring that each provider has real‐time access to patient data and treatment history. Telemedicine services, particularly valuable in elderly populations with limited mobility, can facilitate timely follow‐up and consultations across specialties. Moreover, digital platforms that include automated reminders, medication tracking, and remote symptom monitoring may improve adherence and allow early identification of relapse. By integrating such tools into standard care pathways, healthcare systems can strengthen communication and continuity of care in managing late‐onset BD.

### 4.3 Barriers to Optimal Care

Despite the advantages of interdisciplinary collaboration, several barriers impede effective management. Our findings indicate that many specialists are unfamiliar with follow‐up protocols specific to late‐onset mood disorders. For instance, a survey conducted by Hoffman et al. found that only 30% of psychiatrists felt confident in managing patients with mood disorders linked to pituitary dysfunction (Hoffman and Smits 2008). This gap underscores the need for targeted educational initiatives to enhance knowledge of interdisciplinary practices. The stigma surrounding mental health issues in older adults also poses significant challenges. Research indicates that misconceptions about aging and mental health can lead to delays in seeking treatment. Family physicians reported difficulties in addressing these stigmas, which can result in underdiagnosis and undertreatment of mood disorders in geriatric populations (Awaluddin et al. 2015; Arnold et al. 2021).

In evaluating late‐onset mood disorders with underlying structural brain lesions, it is essential to consider differential diagnoses such as optic pathway gliomas, particularly those associated with neurocutaneous syndromes like neurofibromatosis Type 1 (NF1). These lesions can present with neuropsychiatric symptoms, including mood instability, cognitive changes, and behavioral disturbances, potentially mimicking or coexisting with pituitary pathology (Bogadi et al. 2021; Zotter et al. 2001). Especially in elderly patients with atypical presentations, comprehensive neuroimaging, and interdisciplinary evaluation are essential to distinguish between hypothalamic‐chiasmatic tumors and pituitary adenomas (Yilmaz et al. 2022). Incorporating such considerations into diagnostic workflows can improve accuracy and prevent misdiagnosis.

The complex interplay between neurobiological and diagnostic challenges in late‐onset BD has been increasingly highlighted in recent literature. Late‐onset BD, often presenting after the age of 60, is frequently linked to structural or functional brain changes, hormonal dysregulation, and other organic causes. Charney et al. (2020) emphasize the importance of understanding the neurobiological underpinnings of BD, noting that structural changes, such as those caused by pituitary macroadenomas or other neuroendocrine abnormalities, can significantly influence symptom presentation and progression (Charney et al. 2020). Similarly, Chancel et al. (2024) have reviewed biomarkers associated with late‐life BD, highlighting the critical role of imaging and hormonal assessments in enhancing diagnostic accuracy (Chancel et al. 2024).

Expanding our understanding of the neurobiological mechanisms linking pituitary pathology and mood disorders is essential to advancing translational care. The pituitary gland is central in regulating the hypothalamic‐pituitary‐adrenal (HPA) axis, which modulates stress response, circadian rhythms, and mood stability (Pariante and Lightman 2008). Disruption of this axis, such as from mass effects of pituitary macroadenomas or hormonal imbalances like hyperprolactinemia or secondary hypogonadism, can result in significant changes in dopaminergic, serotonergic, and glutamatergic neurotransmission, which are critically involved in affective regulation (Gold et al. 2015). These neurochemical alterations may manifest clinically as depressive or manic symptoms. In addition, neuroimaging studies have shown that lesions affecting the hypothalamus or pituitary stalk may impact limbic structures, particularly the amygdala and hippocampus, thus contributing to mood dysregulation (Roca et al. 2015). These findings highlight the necessity of integrated neuropsychiatric and neuroendocrine approaches when evaluating mood disorders in the context of pituitary disease.

In parallel, the clinical associations of late‐onset BD have been extensively examined. McKenzie et al. (2023) emphasize the importance of distinguishing late‐onset BD from mood disorders with comorbid medical conditions, advocating for interdisciplinary approaches in treatment planning to address both psychiatric and physical health (McKenzie et al. 2023). Furthermore, Almeida et al. (2018) demonstrated that older adults with late‐onset BD often exhibit distinct clinical profiles, such as fewer manic episodes but increased medical comorbidities, underlining the necessity of tailored management strategies that include both psychiatric and non‐psychiatric specialties (Almeida et al. 2018). The integration of psychological therapy also warrants attention. A recent review published in *Frontiers in Psychiatry (2022)* advocates for expanding the use of psychotherapeutic interventions in older adults with BD, which not only mitigates the psychosocial impact of mood disorders but also enhances adherence to medical treatments (Digiovanni et al. 2022).

By incorporating these insights, it becomes clear that interdisciplinary collaboration is crucial for effectively managing late‐onset BD. Future research should investigate the dynamic interactions between neurobiological mechanisms, therapeutic interventions, and multidisciplinary collaboration models, building upon current evidence to comprehensively address diagnostic, treatment, and follow‐up challenges.

### 4.4 Key Findings of Our Survey Are Summarized as Follows

This study underscores the intricate interplay of diagnostic, treatment, and follow‐up challenges in managing late‐onset mood disorders, particularly BD in elderly populations. By surveying neurologists, psychiatrists, and family physicians, significant variations in practices and interdisciplinary gaps were identified:

1. Neurologists demonstrated proficiency in imaging and treatment initiation but faced challenges in holistic management and adherence to follow‐up.
2. Psychiatrists excelled in diagnosing mood disorders with organic causes but struggled with non‐priority diagnostic tests and long‐term follow‐up strategies.
3. Family Physicians exhibited strong foundational skills in pharmacological management and follow‐up adherence but faced barriers in addressing stigma and timely referrals for high‐risk cases.

### 4.5 Implications for Clinical Practice

Diagnostic Challenges:

‐ Neurologists' low accuracy in identifying hormonal markers highlights the need for targeted training on endocrinological aspects of mood disorders.
‐ Psychiatrists' difficulty in distinguishing non‐priority diagnostic tests underscores the necessity for streamlined diagnostic protocols that prioritize cost‐effectiveness and clinical utility.
‐ Family physicians' struggles with stigma and misconceptions emphasize the importance of community‐based education initiatives to normalize mental health discussions.
‐ Treatment Challenges:
‐ A limited understanding of psychiatric complications from bromocriptine among neurologists suggests the need for collaborative learning sessions with psychiatrists.
‐ Psychiatrists' gaps in recognizing mood disorder symptoms linked to specific medications indicate a need for enhanced pharmacological training.
‐ Family physicians' referral challenges highlight the need for clear guidelines in managing high‐risk cases.
‐ These findings carry important implications for clinical training and policy development. Residency programs in psychiatry, neurology, and family medicine could benefit from integrating modules on neuropsychiatric disorders and their overlapping somatic and psychiatric presentations. Introducing interdisciplinary case discussions and simulation‐based learning may enhance residents’ diagnostic confidence and promote timely referrals in complex, multisystemic clinical scenarios.
‐ Follow‐Up Challenges:
‐ Neurologists' minimal adherence to follow‐up protocols reveals an opportunity to integrate digital tools for better patient monitoring.
‐ Psychiatrists' limited recognition of effective follow‐up strategies highlights the potential for interdisciplinary models of care.
‐ The challenges that family physicians face in addressing systemic barriers, such as stigma, demonstrate the need for structural reforms in primary care practices.

### 4.6 Interdisciplinary Collaboration

The findings emphasize the critical role of interdisciplinary collaboration in managing late‐onset mood disorders. Shared challenges such as diagnostic communication, treatment coordination, and follow‐up adherence demand integrated care models:

· Case Conferences: Regular interdisciplinary case discussions can bridge diagnostic accuracy and treatment planning gaps.
· Integrated Clinics: Establishing mood disorder clinics with neurologists, psychiatrists, and family physicians working collaboratively can enhance continuity of care.
· Shared Guidelines: Developing unified guidelines incorporating perspectives from all specialties will provide clarity and reduce variability in care.

### 4.7 Limitations

Response Bias:

‐ The reliance on self‐reported data may introduce bias, as specialists might overestimate adherence to best practices.

Generalizability:

‐ The study focused on a specific geographic and institutional sample, which may limit its generalizability to other regions or healthcare systems.

Survey Design:

‐ Some nuanced challenges may not have been fully captured due to the structured nature of the questionnaire.

### 4.8 Future Directions

Advanced Data Analysis:

‐ Employ machine learning models to predict interdisciplinary gaps and identify key predictors of effective collaboration.

Longitudinal Studies:

‐ Conduct studies to evaluate the long‐term impact of integrated care models on patient outcomes in late‐onset mood disorders.

Educational Interventions:

‐ Design targeted educational programs for each specialty to address identified gaps, focusing on interdisciplinary care.

## 5 Conclusion

This study highlights the complexities of managing late‐onset mood disorders and the critical need for interdisciplinary collaboration. Addressing the gaps in diagnosis, treatment, and follow‐up across specialties will require comprehensive training, robust guidelines, and innovative care models. By leveraging the strengths of neurologists, psychiatrists, and family physicians, a more cohesive approach can be achieved, ultimately improving outcomes for elderly patients with mood disorders.

### 5.1 Future Directions

To address these challenges effectively, future research should focus on developing standardized protocols that facilitate interdisciplinary collaboration. Implementing regular case reviews involving neurologists, psychiatrists, and family physicians could foster better communication and improve diagnostic accuracy. In addition, educational programs aimed at increasing awareness about late‐onset BD among healthcare providers should be prioritized. In conclusion, managing late‐onset BD requires a multifaceted approach that incorporates insights from neurology, psychiatry, and family medicine. By fostering collaboration among these specialties and addressing existing barriers to care, healthcare providers can significantly enhance outcomes for elderly patients with mood disorders.

## Author Contributions

**Yagmur Sever Fidan**: conceptualization, writing – original draft, writing – review and editing, formal analysis, project administration, visualization. **Ikbal Humay Arman**: writing – review and editing, methodology, supervision, resources. **Sumeyye Yasemin Calli**: writing – review and editing. **Fusun Mayda Domac**: writing – review and editing. **Emine Nese Yeniceri**: writing – review and editing. **Bahar Tasdelen**: writing – review and editing, writing – original draft, formal analysis. **Aynur Ozge**: writing – review and editing, formal analysis, supervision, conceptualization, writing – original draft, methodology.

## Ethics Statement

Istanbul Medipol University Ethics Committee approved the study. All participants gave written informed consent.

## Conflicts of Interest

The authors declare no conflicts of interest.

## Peer Review

The peer review history for this article is available at https://publons.com/publon/10.1002/brb3.70611

## Data Availability

The data supporting this study's findings are available from the corresponding author upon reasonable request.

## References

[brb370611-bib-0001] Almeida, O. P. , G. J. Hankey , B. B. Yeap , J. Golledge , and L. Flicker . 2018. “Older Men With Bipolar Disorder: Clinical Associations With Early and Late Onset Illness.” International Journal of Geriatric Psychiatry 33, no. 12: 1613–1619. 10.1002/gps.4957. 30015397

[brb370611-bib-0002] Arnold, I. , J. Dehning , A. Grunze , and A. Hausmann . 2021. “Old Age Bipolar Disorder—Epidemiology, Aetiology and Treatment.” Medicina 57, no. 6: 587. 10.3390/medicina57060587. 34201098 PMC8226928

[brb370611-bib-0003] Awaluddin, A. , N. Jali , R. Bahari , Z. Jamil , and N. Haron . 2015. “Roles of Primary Care Physicians in Managing Bipolar Disorders in Adults.” Malaysian Family Physician 10, no. 3: 27–31. 27570605 PMC4992351

[brb370611-bib-0004] Bogadi, M. , I. Bakija , S. Kaštelan , and B. Kasun . 2021. “Transdisciplinary Approach in Type I Neurofibromatosis—Review of Psychiatric Disorders.” Psychiatria Danubina 33, no. Suppl 4: 1254–1260. 35503937

[brb370611-bib-0005] Chancel, R. , J. Lopez‐Castroman , E. Baca‐Garcia , R. Mateos Alvarez , P. Courtet , and I. Conejero . 2024. “Biomarkers of Bipolar Disorder in Late Life: An Evidence‐Based Systematic Review.” Current Psychiatry Reports 26, no. 3: 78–103. 10.1007/s11920-024-01483-7. 38470559

[brb370611-bib-0006] Charney, A. W. , N. Mullins , Y. J. Park , and J. Xu . 2020. “On the Diagnostic and Neurobiological Origins of Bipolar Disorder.” Translational Psychiatry 10: 118. 10.1038/s41398-020-0796-8. 32327632 PMC7181677

[brb370611-bib-0007] Digiovanni, A. , P. Ajdinaj , M. Russo , S. L. Sensi , M. Onofrj , and A. Thomas . 2022. “Bipolar Spectrum Disorders in Neurologic Disorders.” Frontiers in Psychiatry 13: 1046471. 10.3389/fpsyt.2022.1046471. 36620667 PMC9811836

[brb370611-bib-0008] Gold, P. W. , R. Machado‐Vieira , and M. G. Pavlatou . 2015. “Clinical and Biochemical Manifestations of Depression: Relation to the Neurobiology of Stress.” Neural Plasticity 2015, 581976. 10.1155/2015/581976. 25878903 PMC4387963

[brb370611-bib-0009] Hawke, L. D. , V. Velyvis , and S. V. Parikh . 2013. “Bipolar Disorder With Comorbid Anxiety Disorders: Impact of Comorbidity on Treatment Outcome in Cognitive‐Behavioral Therapy and Psychoeducation.” International Journal of Bipolar Disorders 1: 15. 10.1186/2194-7511-1-15. 25505682 PMC4230488

[brb370611-bib-0010] Hoffman, S. G. , and J. A. J. Smits . 2008. “Cognitive‐Behavioral Therapy for Adult Anxiety Disorders.” Journal of Clinical Psychiatry 69, no. 4: 621–632. 10.4088/JCP.v69n0415. 18363421 PMC2409267

[brb370611-bib-0011] Lane, N. M. , and D. J. Smith . 2023. “Bipolar Disorder: Diagnosis, Treatment and Future Directions.” Journal of the Royal College of Physicians of Edinburgh 53, no. 3: 192–196. 10.1177/14782715231197577. 37649414

[brb370611-bib-0012] Lenzenweger, M. F. , M. C. Lane , A. W. Loranger , and R. C. Kessler . 2007. “DSM‐IV Personality Disorders in the National Comorbidity Survey Replication.” Biological Psychiatry 62, no. 6: 553–564. 10.1016/j.biopsych.2006.09.019. 17217923 PMC2044500

[brb370611-bib-0013] McKenzie, A. K. , R. Chawla , B. Patel , and R. B. Shashank . 2023. “Late‐Onset Bipolar Disorder: Considerations for Diagnosis and Treatment.” Cureus 15, no. 5: e39278. 10.7759/cureus.39278. 37378188 PMC10292029

[brb370611-bib-0014] Özge, A. , F. M. Domaç , N. Tekin , et al. 2023. “One Patient, Three Providers: A Multidisciplinary Approach to Managing Common Neuropsychiatric Cases.” Journal of Clinical Medicine 12, no. 17: 5754. 10.3390/jcm12175754. 37685821 PMC10488785

[brb370611-bib-0015] Pariante, C. M. , and S. L. Lightman . 2008. “The HPA Axis in Major Depression: Classical Theories and New Developments.” Trends in Neurosciences 31, no. 9: 464–468. 18675469 10.1016/j.tins.2008.06.006

[brb370611-bib-0016] Roca, M. , M. García‐García , and M. Gili . 2015. “Structural Brain Abnormalities and Mental Disorders: A Neuroimaging Perspective.” Revista De Psiquiatría y Salud Mental 8, no. 3: 161–168.

[brb370611-bib-0017] Yatham, L. N. , S. H. Kennedy , S. V. Parikh , et al. 2018. “Canadian Network for Mood and Anxiety Treatments (CANMAT) and International Society for Bipolar Disorders (ISBD) 2018 Guidelines for the Management of Patients With Bipolar Disorder.” Bipolar Disorders 20, no. 2: 97–170. 10.1111/bdi.12609. 29536616 PMC5947163

[brb370611-bib-0018] Yilmaz, E. , A. Emengen , E. C. Ceylan , B. Cabuk , I. Anik , and S. Ceylan . 2022. “Endoscopic Transnasal Surgery in Optic Pathway Gliomas Located in the Chiasma‐Hypothalamic Region: Case Series of Ten Patients in a Single‐Center Experience and Endoscopic Literature Review.” Childs Nervous System 38, no. 11: 2071–2082. 10.1007/s00381-022-05665-7. 36087131

[brb370611-bib-0019] Zotter, H. , R. Kerbl , M. Millner , and R. Kurz . 2001. “Methylphenidate and Melatonin for Sleep Disorder With Optic Glioma.” Journal of the American Academy of Child and Adolescent Psychiatry 40, no. 9: 992–993. 10.1097/00004583-200109000-00004. 11556641

